# The origin, function, and diagnostic potential of RNA within extracellular vesicles present in human biological fluids

**DOI:** 10.3389/fgene.2013.00142

**Published:** 2013-07-30

**Authors:** Douglas D. Taylor, Cicek Gercel-Taylor

**Affiliations:** Department of Obstetrics, Gynecology, and Women's Health, University of Louisville School of MedicineLouisville, KY, USA

**Keywords:** exosomes, microvesicles, transcriptome, microRNA, early diagnosis

## Abstract

We have previously demonstrated that tumor cells release membranous structures into their extracellular environment, which are termed exosomes, microvesicles or extracellular vesicles depending on specific characteristics, including size, composition and biogenesis pathway. These cell-derived vesicles can exhibit an array of proteins, lipids and nucleic acids derived from the originating tumor. This review focuses of the transcriptome (RNA) of these extracellular vesicles. Based on current data, these vesicular components play essential roles as conveyers of intercellular communication and mediators of many of the pathological conditions associated with cancer development, progression and therapeutic failures. These extracellular vesicles express components responsible for angiogenesis promotion, stromal remodeling, signal pathway activation through growth factor/receptor transfer, chemoresistance, and genetic exchange. These tumor-derived extracellular vesicles not only to represent a central mediator of the tumor microenvironment, but their presence in the peripheral circulation may serve as a surrogate for tumor biopsies, enabling real-time diagnosis and disease monitoring.

## Background

The release of 50–200 nm sized membranous vesicles into biological fluids by viable tumor cells was initially described by us over three decades ago (Taylor and Doellgast, 1979) and has since been demonstrated multiple cell types and systems. *In vivo*, these nano-sized vesicles released by tumor cells accumulate in biologic fluids, including blood,urine, ascites, and pleural fluids (Taylor and Gercel-Taylor, 2011). The release and accumulation of these extracellular vesicles appear to be important in the malignant transformation process. Extracellular vesicles have been identified by various terms, including high molecular weight complexes, membrane fragments, exosomes, microvesicles, microparticles, and extracellular vesicles. While more recently restrictive definitions have been applied to these cell-derived vesicles, significant overlap (in terms of size, markers, cargoes, and function) exists between structures identified as exosomes and microvesicles. Within the circulation, it may not be possible to differentiate 50–100 nm “exosomes” from 50 to 200 nm “microvesicles.” In many studies, uncharacterized cell-derived vesicles (in terms of markers or size) are termed “microvesicles,” while numerous studies define “exosomes” solely based on density and the presence of the cell surface markers, tetraspanins. These overlaps in vesicle properties and terms suggest these distinctions may not be clear-cut. For these reasons, this review uses the term “extracellular vesicles” to include all 50–200 nm tumor-derived vesicles.

The “exosome” term was coined in 1981 for “exfoliated membrane vesicles with 5′-nucleotidase activity” (Trams et al., 1981). This term, “exosome,” originated from the discovery of neoplastic cell line-derived exfoliated vesicles, which mirrored the 5′-nucleotidase activity of the parent cells (Trams et al., 1981). Several years later, vesicles release via the canonical pathway upon multi-vesicular endosome fusion with the cell surface was demonstrated in cultured sheep (Pan et al., 1983) and rat (Harding et al., 1983) reticulocytes. After purification by ultracentrifugation, the pelleted vesicles were found to contain the transferrin receptor that was also found in native reticulocytes (Johnstone et al., 1987). These reports proposed that this represented a mechanism for the elimination of specific cellular components as the reticulocytes matured and differentiated. These cell-derived vesicles were redefined as “exosomes” to differentiate them from “endosomes.” The disparate natures of these studies are reflected in the various names that were proposed and which are still used to identify the cell surface-released and endocytic vesicles of different origins. It is of noted that these later studies (Harding et al., 1983; Pan et al., 1983; Johnstone et al., 1987) were exclusively *in vitro*.

The *in vivo* appearance of tumor-derived vesicles within the circulation was initially demonstrated in ovarian cancer patients (Taylor and Doellgast, 1979; Taylor et al., 1980, 1983). These gynecologic cancer patients exhibited intact extracellular vesicles within their peripheral circulation and malignant effusions (ascites and cyst fluids). These tumor-derived extracellular vesicles were found to express molecular markers that were generally linked with tumor plasma membranes, including placental type alkaline phosphatase and mdr-1 (Taylor et al., 1985, 1989; Taylor and Black, 1986); however, proteins not generally associated with plasma membranes, such as p53, GRP78 and nucleophosmin, were also identified with these circulating vesicles (Chinni et al., 1997; Manahan et al., 2001).

Extracellular vesicles have since been demonstrated to be released by a variety of non-cancerous cells, particularly cells of the immune system, including dendritic cells, macrophages, B cells, T cells, and NK cells (Théry et al., 2009), as well as embryonic cells (Atay et al., 2011a,b). These extracellular vesicles have been demonstrated to be key mediators/regulators of normal immune responses (Whiteside, 2013). One can view tumors as a “cyber-terrorist” using these extracellular vesicles to elicit aberrant immune regulation. Extracellular vesicles released by the tumor may elicit a tolerogenic response and participate in other immune mechanisms, such as platelet activation, mast cell degranulation, germinal center reaction, and potential engulfment of apoptotic cells. The aberrant release of extracellular vesicles by tumors may allow them to circumvent these immunoregulatory antigen delivery pathways and evade immunosurveillance (Taylor and Black, 1986; Taylor and Gercel-Taylor, 2011).

## Extracellular vesicles composition and characterization

Over the past three decades, shed tumor-derived vesicles have been characterized for multiple human cancer types and they are not exact replicates of the plasma membrane or other membranous compartments of the originating tumor cells, but they represent “micromaps” with enhanced expression of tumor antigens, as well as other macromolecules, including major histocompatibility antigens (Taylor and Gercel-Taylor, 2011). Exosomes, vs. microvesicles, have been defined, based on size (30–100 nm lipid bilayer vesicles), density (1.12 g/ml for B cell derived to 1.19 g/ml for intestinal cell derived) and expression of specific biomarkers (including tetraspanins) (Théry et al., 2009). Extracellular vesicles can be viewed as cytoplasm enclosed in a lipid layer with the external domains of transmembrane proteins exposed to the extracellular environment in their normal cellular orientation. Electron microscopic studies have demonstrated the fusion of the limiting membrane of MVB with the plasma membrane as well as release of ILVs in different cell types of hematopoietic origin, such as Epstein-Barr virus (EBV)-transformed B cells (Zumaquero et al., 2010), mastocytes (Admyre et al., 2008), dendritic cells (Montecalvo et al., 2008), platelets (Rak, 2010), macrophages (Anand, 2010) and cells of non-hematopoietic origin like neurons and epithelial cells (Kesimer et al., 2009).

The exact mechanisms by which cells release exosomes/microvesicles remain unclear; however, the release is modulated by extracellular signals (Ostrowski et al., 2010). Three primary mechanisms have been proposed for the release of cellular components into the extracellular space: (1) exocytic fusion of multivesicular bodies (MVBs) resulting in “exosomes,” (2) budding of vesicles directly from the plasma membranes resulting in shed “microvesicles” and (3) cell death leading to apoptotic bodies. The first two mechanisms are properties of viable cells and are energy requiring events. While most isolation protocols readily exclude apoptotic bodies, current methods do not differentiate extracellular vesicles from the endocytic pathway from shed “microvesicles” from the plasma membrane. As a result, most studies on these extracellular vesicle populations include a mixture of exosomes and microvesicles, which may confuse interpretation of biochemical data.

Since the formation of exosomes has an endocytic origin, this mechanism is a process of the endosomal pathway, including endocytic vesicles, early endosomes, late endosomes and lysosomes. The endocytic vesicles are formed through either clathrin- or non-clathrin-mediated endocytosis at the plasma membrane and are transported to early endosomes (Yu et al., 2006; Valapala and Vishwanatha, 2011). The late endosomes develop from early endosomes via acidification, changes in their protein content and their ability to fuse with vesicles or other cellular membranes. Early endosomes are localized near the outer margin of the cells and exhibit a tubular appearance, in contrast late endosomes are localized proximal to the nucleus and are spherical in shape. An essential step in MVB formation from late endosomes is reversed budding. In this step, limiting membranes of late endosomes “bud” into their lumen, resulting in a continuous enrichment of internal luminal vesicles (Yu et al., 2006). MVBs have been demonstrated to be involved in the exocytic fusion of their external membrane with the plasma membrane of the cell, resulting in release of their segregated vesicles to the extracellular space. Within the vesicles, two invaginations occur, such that the membrane orientation of proteins within the vesicles is the same as the plasma membrane of the cell. The release of large biomolecules through the plasma membrane can occur through the process termed exocytosis, which has regulatory and signaling functions. Exocytosis can be either constitutive (non-calcium-triggered) or regulated (calcium-triggered) (Yu et al., 2006; Valapala and Vishwanatha, 2011). Constitutive exocytosis occurs in all cells and serves to secrete extracellular matrix components or to incorporate newly-synthesized proteins into the plasma membrane following fusion with transport vesicles. Regulated exocytosis is critical to events, such as neurologic signaling, as synaptic vesicles fuse with the membrane at the synaptic cleft (Graner et al., 2009).

Extracellular vesicles isolated from the extracellular environment of tumors (such as from the peripheral circulation), either *in vitro* or *in vivo*, exhibit overlapping similarities in size (defined by dynamic light scattering), morphology (defined by electron microscopy), density (defined by of sucrose gradient centrifugation), and protein markers of both the endosomes and plasma membranes (defined by western immunoblotting and mass spectrometry) (Graner et al., 2009; Mathivanan and Simpson, 2009; Xiao et al., 2012). While many of the definitions are still used, we now recognize their flaws (Table 1). The apparent size and shape of exosomes appear to be artifacts of fixation and drying associated with electron microscopy. Principal markers of exosomes are tetraspanins, which as plasma membrane associated components are present on most vesicles, regardless for their origin. The importance of the endocytic pathway of vesicle formation has also been questioned as knock-out studies with Rab proteins only diminished vesicle release by ~30% (based on exosomal protein) (Peinado et al., 2012).

**Table 1:** **Glossary of extracellular vesicle terms**.

**Term**	**Current definition (Théry et al., 2009)**	**Deficiencies**
Exosomes	Size: 50–100 nm Shape: Cup shaped Sedimentation: 100,000 × g Markers: Tetraspanins (CD63/CD9), Alix, TSG101, ESCRT Lipids: Cholesterol, sphingomyelin, ceramide, lipid rafts, phosphatidylserine Origin: Multivesicular endosomes	Size and shape may be altered by the fixation process Tetraspanins are also plasma membrane markers and are not specific for vesicles derived from MVB Knockout studies suggest MVB-derived vesicles represent only a portion of the 50–200 nm vesicles Lipid composition is also shared with “micromaps” of the plasma membrane
Microvesicles	Size: 100–1000 nm Shape: Irregular Sedimentation: 100,000 × g or 10,000 × g or 1200 × g (dependent on size) Markers: Intergins, selectins, CD40 ligand Lipids: Phosphatidylserine Origin: Plasma membrane	Most circulating vesicles fall in the range of 50–200 nm. *In vivo*, larger vesicles are generally not observed Impossible to distinguish 50–200 nm microvesicles from exosomes based on sedimentation Microvesicles derived from surface “micromaps” shared lipid and protein compositions with exosomes Tetraspanins can be detected on vesicles of all size ranges Microvesicle- “specific” markers were demonstrated on vesicles derived from B cells, thus these may not be relevant to those derived from other cell types
Apoptotic bodies	Size: 400–1000 nm Shape: Heterogeneous Sedimentation: 100,000 × g or 10,000 × g or 1200 × g Markers: Histones Lipids: Not determined Origin: Plasma membrane	Exosomes and microvesicles are the products of viable, healthy, proliferating cells, while apoptotic bodies are released as part of apoptotic cell death Exosomes and microvesicles exhibit high degrees of selectivity in cargoes, while apoptotic bodies contain debris of the dying cell

**Figure 1:**
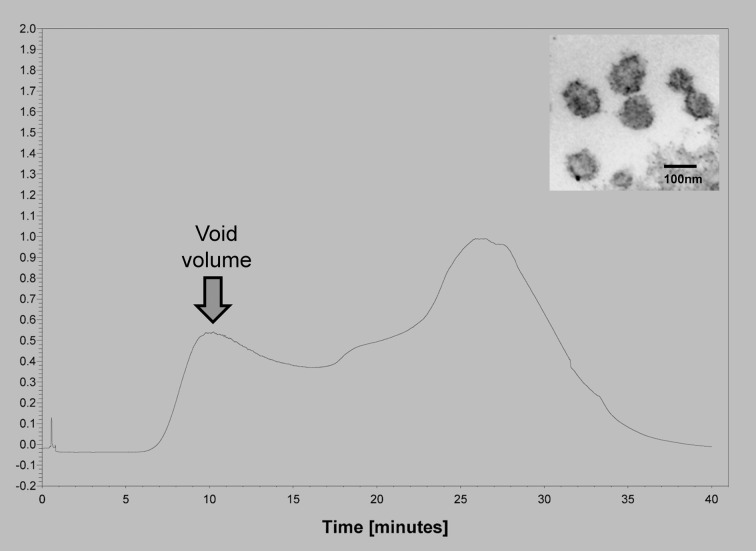
**Example of the isolation of extracellular vesicles from an ovarian cancer patients using size exclusion chromatography on Sepharose 2B.** Insert shows an electron micrograph of the void volume material, demonstrating the presence of 50–150 nm vesicles.

We have compared the extracellular vesicle populations obtained from biologic fluids of ovarian cancer patients by both the technique described to isolate extracellular vesicles and our original chromatographic method isolating “microvesicles” (Taylor and Gercel-Taylor, 2005) (Figure 1). This comparative study demonstrated that these *in vivo* derived vesicles from both techniques isolated cup-shaped vesicles, with a density between 1.13 and 1.17 g/ml, a diameter between 50 and 150 nm, and expressing CD63, Alix, VPS35, galectin 3, HSP90, fibronectin, and placental alkaline phosphatase (Taylor and Gercel-Taylor, 2005; Taylor et al., 2011). While patient-derived circulating extracellular vesicles fit within the definition of exosomes, the contribution of these two populations is unclear. Since both populations exist within the peripheral circulation of cancer patients, this distinction between exosomes and shed microvesicles may not be critical to understand the biologic activities of these vesicles as they can interact with target cells of the host as a mixture. In a subsequent study comparing methods, we further described a more uniform vesicle size and superior recovery of vesicle components (proteins and RNA) by chromatography vs. centrifugation (Taylor et al., 2011). This advantage of the chromatographic approach may relate to the fact that vesicles are not subjected to shearing force associated with centrifugation.

The increased release of extracellular vesicles and their accumulation appear to be important in the malignant transformation process. Although shedding of extracellular vesicles occurs in other types of cells under specific physiological conditions, the accumulation of extracellular vesicles from tumor cells is augmented in biologic fluids. Recently, circulating vesicles of normal individuals, patients with benign ovarian disease and patients with ovarian cancer have been investigated using the Nanoparticle Tracking Analysis system (NanoSight) (Gercel-Taylor et al., 2012). The presence of circulating vesicular materials was demonstrated in all individuals; however, ovarian cancer patients exhibit ~3–4-fold more vesicular material. In these cancer patients, the size range of these vesicles was between 50 and 250 nm, with the major peak at 98–99 nm.

## Cargoes of extracellular vesicles

Extracellular vesicles contain proteins, non-coding RNAs, and mRNAs, and the exosomal lipid bilayer appears to protect these materials from degradation. While protein and RNA cargoes of extracellular vesicles vary depending on the originating cell, there are conserved proteins among extracellular vesicles from different cellular origins (Mathivanan and Simpson, 2009). The protein composition of extracellular vesicles has been extensively analyzed by various techniques including Western blotting, fluorescence-activated cell sorting, immuno-electron microscopy and mass spectrometry. All extracellular vesicles exhibit cytoskeleton proteins (such as ezrin and actins), proteins associated with the MVB biogenesis (such as alix and TSG101), membrane transport and fusion proteins (e.g., annexins and Rab proteins), and tetraspanins (e.g., CD9, CD63, and CD81). A catalog of proteins, RNAs, and lipids assoicated with extracellular vesicles can be found at www.exocarta.org. Currently, ExoCarta lists entries for 13,333 proteins, 2375 mRNAs, 764 microRNA, and 194 lipids associated with extracellular vesicles.

We have analyzed the patient-derived exosomal proteome using ion trap mass spectrometry and identified 232 unique proteins. These proteins were classified as percent of the identified total proteins into molecular chaperones (8.5%), vesicle fusion (8.5%), cytoskeletal proteins and proteins involved in the assembly/disassembly of the cytoskeletal networks (17.6%), anionic and cationic ion transport channels (3.7%), proteins involved in lipid (6.9%), carbohydrate (3.2%) and amino-acids (2.1%) metabolisms, proteins involved in DNA replication (6.9%), mRNA splicing (5.3%), transcription/translation (5.3%), post-transcriptional protein modification (13.8%), and signal transduction (2.7%). Our studies demonstrated that cytosolic proteins were highly represented and we observed a diverse array of cytoskeletal constituents (actin, α-actinin-1, cofilin, filamin-A-B-C, tubulins, gelsolin, profilin-1, spectrin, symplekin, talin, vinculin, myosins). We identified that transmembrane proteins were also abundant, including multiple integrins (β 1, α 3, α v), intercellular adhesion molecule 1 (ICAM-1), and mucin-4. A variety of channels were observed, such as the voltage-dependent anion-selective channel protein 2 and 3, chloride intracellular channel protein 1, sodium/potassium-transporting ATPase subunit β-3, long of sodium/potassium-transporting ATPase subunit α-1, and transitional endoplasmic reticulum ATPase. In line with their endocytic origin, exosomal proteins belonging to the ESCRT complex that are important protein complexes involved in ubiquitin-dependent exosome biogenesis, have also observed (Doring et al., 2010). These ESCRT-associated proteins include vacuolar protein sorting-associated protein 35 (VPS-35), Alix, ubiquitin-like modifier-activating enzyme and ubiquitin carboxyl-terminal hydrolases. We demonstrated that proteins involved in membrane trafficking and fusion processes were enriched (annexin A2, A5, A6, clathrin heavy chain 1/2, coatomer subunit β, Rab1b, Rab2a, and Rab7a). A group of markers of endosomes and lysosomes were also detected (cathepsin-C, D, EH domain-containing protein 1 and β-hexoaminidase), and several chaperonnes were identified (HSP70, HSP90, HSC70, HSPA4, 8, 9, HSPA1A/B, HSPB1, HSP47, HSPA5, HSPβ 1, HSPD1, HSP90AB1, B1, AA1; T-complex protein 1, endoplasmin, and protein disulfide-isomerase A3, A4, A6) (Graner et al., 2009; Mathivanan and Simpson, 2009).

## Extracellular vesicle-associated RNA

The current hypothesis for the stability of circulating RNA is that they are released from cells in membranous vesicles. Recent data confirm that extracellular RNA can exist in four forms: free RNA, Argonaut 2-bound RNA, high-density lipoprotein-bound RNA and vesicle-associated RNA. This review focuses on RNA associated with extracellular vesicles. These extracellular vesicles are generated constitutively by most, but not all, cell types and contain both mRNAs and non-coding RNA. The ability of extracellular vesicles to transfer genetic information may facilitate cancer spread by delivering genetic material and oncogenic proteins. RNA profiles of extracellular vesicles differ from that of cellular RNA, since vesicles contain primarily small RNA, such as mRNA and microRNA, in the absence of ribosomal RNA (Skog et al., 2008; Taylor and Gercel-Taylor, 2008).

The presences of circulating RNAs have been extensively investigated, despite the presence of highly stable RNases, which should degrade any free RNA. The majority of the circulating RNAs have been defined as microRNAs based on the molecular weight (Mitchell et al., 2008). Studies also demonstrated that microRNAs not only have high stability in body fluids, but also survive in the unfavorable physiological conditions such as freeze-thawing, extreme variations in pH and long time at room temperature (Mitchell et al., 2008; Duggagupta et al., 2011; Chen et al., 2012). Whereas adding detergents, such as Triton X or SDS, to serum or plasma makes microRNAs easily degradation by RNases (Zhang et al., 2010). The results indicate there are at least two approaches responsible for the stability of extracellular microRNAs: be packaged in membrane-encapsulated vesicle and be protected by RNA-binding proteins.

The stability of extracellular microRNAs has been hypothesized to be due to the formation of the RNA-vesicle. During RISC disassembly in the cytoplasm, some microRNAs are found to be sorted into MVBs, which are commonly considered to form exosomes by fusion with the plasma membrane (Simons and Raposo, 2009). Both exosome and microvesicle can easily translocate across the cell membrane, which makes microRNAs enter recipient cells easily and mediate cell-to-cell communication.

Our studies have indicated that many of RNAs enriched in the extracellular vesicles may not be abundant, or even detectable, in the originating cell or highly expressed within the cell and low or absent within extracellular vesicles (Gercel-Taylor et al., 2013), indicating sorting of specific RNAs into extracellular vesicles. These released microRNAs can be classified in three categories based on the ratio between the amount of microRNA released from the cells and the amount retained in the cell (Pigati et al., 2010). The first group is selectively released microRNAs, which are characterized by being primarily released from tumor cells with relatively low concentrations remaining in the cell. In contrast, normal cells do not release appreciable quantities of these microRNAs (Pigati et al., 2010). An additional group of released microRNAs are those released in equal levels as they appear within the cell, termed neutrally released microRNA. These neutrally released microRNAs include miR16 and miR21, where the abundance in extracellular vesicles reflects increased abundance in the tumor cells. The selectivity of release of specific microRNAs differs depending on the cell type (Rabinowits et al., 2009; Pigati et al., 2010; Mittelbrunn et al., 2011). Selectivity appears to be influenced by malignant transformation. Breast and ovarian tumor cells have been demonstrated to release >99% of miR451 and miR1246 produced by the cells (Pigati et al., 2010; Gercel-Taylor et al., 2013). These selectively released microRNAs have been linked to the malignant phenotype. MiR451 has been identified as a tumor suppressor, defining proliferation and cell polarity. miR451 has also been shown to induce chemosensitivity. miR1246 induces p53-dependent apoptosis triggered by DNA damage (Zhang et al., 2011). The changes in the release of cancer-related microRNAs may suggest a role for selective microRNA export in malignant transformation, and it may provide a cancer signature within the exported, circulating microRNA population.

While the mechanism of this selective sorting is unclear, some have postulated this selectivity relates to microRNA/RNA-induced silencing complex (RISC) components. Extracellular vesicles contain components of the microRNA/RISC, such as Argonaut 2, together with several RNA-binding proteins known to regulate RNA traffic between the nucleus and the cytoplasm. It can be therefore hypothesized that, during vesicle biogenesis, these RNA binding proteins regulate the accumulation of selected RNAs within extracellular vesicles. Studies on the transfer of reporter mRNAs and their translation into proteins, demonstrated both *in vitro* and *in vivo*, suggest that the mRNA delivered by extracellular vesicles is functional (El-Andaloussi et al., 2012; Tetta et al., 2013).

Extracellular vesicles derived from other tumors such as colorectal (Silva et al., 2012), lung (Rabinowits et al., 2009), and prostate cancer cells (Bryant et al., 2012) alter the phenotype of normal cells by transferring specific RNA subsets. In contrast, extracellular vesicles released from the surrounding cells may modify cancer cell gene expression (Bryant et al., 2012). Extracellular vesicles derived from cancer stem cells were shown to contain pro-angiogenic RNAs able to induce a pre-metastatic niche in the lungs, whereas those derived from differentiated cancer cells were not able to induce this niche and their mRNA and microRNA content differs (Grange et al., 2011). Extracellular vesicles from cancer stem cells contained miR29a, miR650, and miR151, all associated with tumor invasion and metastases, along with miR19b, miR29c, and miR151, known to be up-regulated in patients with renal carcinomas (Grange et al., 2011).

Extracellular vesicles have been isolated and analyzed from both normal healthy individuals and patients with various physiological conditions. We have previously shown that cancer patients (Gercel-Taylor et al., 2012) and pregnant women (Atay et al., 2011a) exhibit more extracellular vesicles in their blood compared to their normal, healthy counterparts. In pregnant women, the extracellular vesicles are thought to play a role in the maternal-fetal tolerance occurring during pregnancy, as it has been shown that placenta extracellular vesicles suppress T lymphocytes (Atay et al., 2011b).

**Figure 2:**
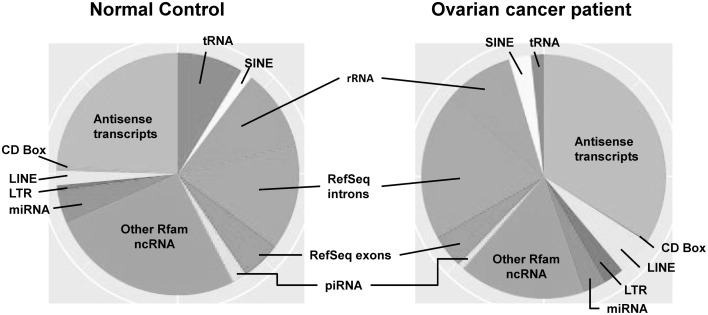
**The distribution of small RNAs derived from circulating extracellular vesicles of normal controls vs. ovarian cancer patients, based on next generation sequencing**.

Most investigations on small RNAs in exosomes have been limited to microRNA; however, next generation sequencing small RNAs in extracellular vesicles is expanding the populations identified. While intracellular microRNAs have been defined in many biological processes, identification of extracellular vesicle-associated microRNAs represents a non-invasive approach to investigate disease-specific microRNA and may provide a method for disease diagnosis (Duggagupta et al., 2011). To detect, analyze, and quantitate the RNA signatures of exosomes derived from biologic fluids, several approaches have been used, including microarrays, quantitative real-time PCR, and next-generation sequencing. The development of high detection sensitivity in next generation sequencing technologies has expanded the identification of the exosomal transcriptome, beyond miRNA. While most studies have focused on exosomal microRNAs, we now recognize the presence of numerous other small RNAs within these circulating exosomes, as well as fragments of larger RNAs (Figure 2). These exosomal small non-coding RNAs are <200 nucleotides in length (generally are 20–30 nt). There are three primary populations of small non-coding RNAs, including siRNAs, miRNAs, and piRNAs. Small non-coding RNAs have been shown to be key regulators in development, apoptosis, stem cell self-renewal, differentiation, and cell integrity maintenance. Piwi-interacting RNAs (piRNAs) are generated from intergenic elements, including transposable elements, through Dicer-independent pathways. These piRNAs function through the Piwi-Argonaute sub-family (AGO3, Aubergine, and Piwi), leading to silencing of transposable elements. A link between piRNAs and cancer has been demonstrated in gastric cancers where two aberrantly expressed piRNAs, piRNA-651 and piRNA-823, were found in gastric tumor tissue vs. paired normal tissue (Cheng et al., 2011, 2012).

## Functions of extracellular vesicles

**Figure 3:**
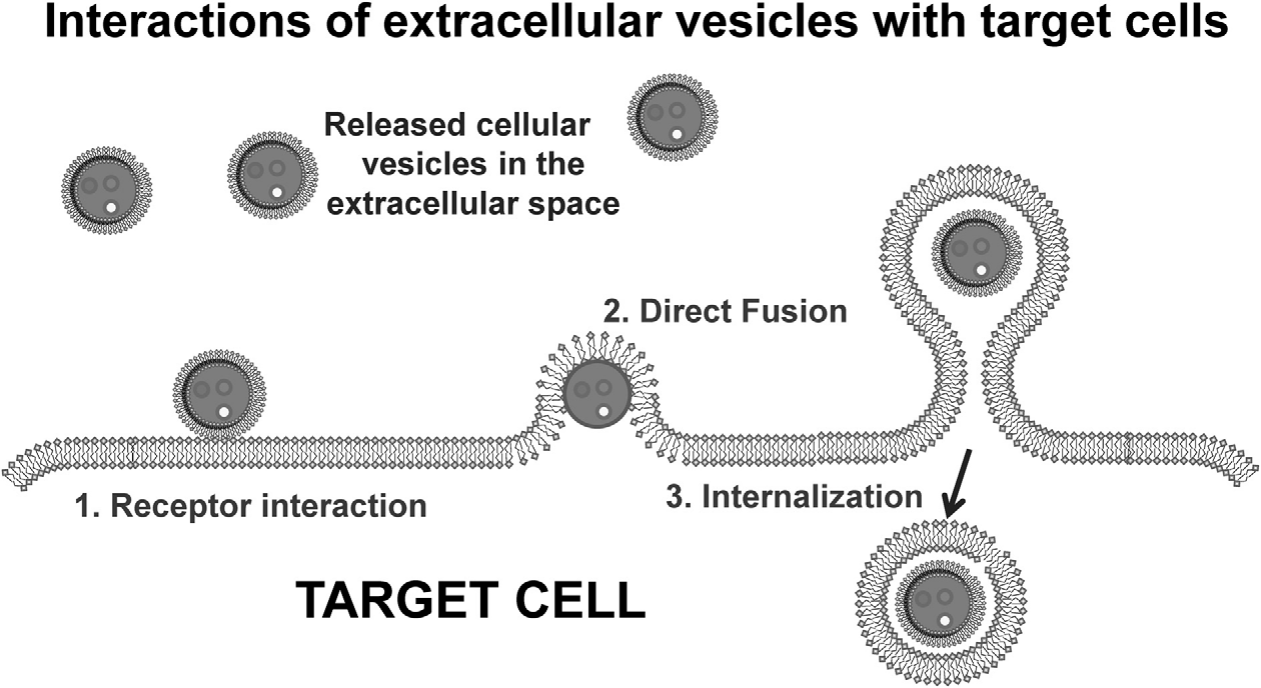
**Scheme of the potential mechanisms of interactions between an extracellular vesicle and a target cells.** The interactions include receptor/ligand binding, fusion, and internalization.

While some early studies implicated extracellular vesicles as “garbage bags” of the cells (Pan et al., 1983; Johnstone et al., 1987), the vesicles released from tumor cells have gained increasing recognition as “vehicles” for intercellular communication. Intercellular communication has been thought to be limited to cell-to-cell adhesion conduits (gap junctions) or secreted signals, such as hormones, neurotransmitters, and cytokines, released from cells and acting in an autocrine or paracrine manner. These extracellular vesicles interact with the plasma membrane of a recipient cell by ligand/receptor binding, fusion or internalization (or a combination of these, Figure 3). If the extracellular vesicles fuse with the target cell, they can transfer their cargo to that recipient cell. Due to the presence of cell-type specific adhesion molecules, extracellular vesicles can interact with specific cells and deliver their “cargoes,” including bioactive lipids, cytokines, growth factors, receptors and genetic materials. In this manner, extracellular vesicles represent a pathway for intercellular transfer of information, similar to that observed with direct cell–cell contact, but that can function at distance. Extracellular vesicles provide stable conformational conditions for their protein content, conserve bioactivity of their proteins, improve bio-distribution and support an efficient interaction with target cells.

The complexity of extracellular vesicle associated bioactive macromolecules supports a critical role in generating the tumor microenvironment (Marhaba et al., 2008; Park et al., 2010). Extracellular vesicles can transfer specific proteins to target cells for the delivery of signaling pathways (Hong et al., 2009; Xiang et al., 2009). The presence of tumor-derived extracellular vesicles can increase matrix metalloproteinase (MMP) secretion and VEGF expression in target cells through the expression of pro-angiogenic molecules, such as members of the tetraspanin family, thereby promoting neo-angiogenesis even at secondary metastatic sites (Nazarenko et al., 2010). The released MMPs can digest the extracellular matrices where they arise. This degradation is enhanced when MMPs are co-released with exosome-associated extracellular MMP inducer (EMMPRIN) (Keller et al., 2009).

Studies have shown that cancer ascites-derived extracellular vesicles carry extracellular matrix-remodeling enzymes, such as metalloproteinases 2 and 9 (MMP-2, MMP-9) (Dolo et al., 1999a,b), and urokinase plasminogen activator (Graves et al., 2004), leading to an increase in extracellular matrix degradation. The expression of matrix-remodeling enzymes increases the tumor's invasive phenotype and promotes metastasis. The presence of pro-angiogenic factors supports neovascularization of the developing tumor. A commonly identified cellular component of the tumor microenvironment is the monocyte/macrophage. Within the microenvironment, tumor-associated macrophages have been shown to assist in tumor progression by expressing cytokine/chemokine profiles that promote angiogenesis, stimulate tumor growth, and elicit immunosuppression by suppressing Th1 responses (Lewis and Pollard, 2006; Whiteside, 2013). The tumor microenvironment is characterized by pro-inflammatory profiles, including interleukin-1β (IL-1β). This profile is generally produced by infiltrating macrophages following interactions with tumors or their components. While we proposed that extracellular vesicles could “educate” macrophages to produce pro-inflammatory cytokines following internalization, we recently demonstrated that the induction of IL-1β was observed even when internalization of vesicles was blocked (Atay et al., 2011a,b). RGD peptides, which are used to block fibronectin binding to macrophage α5β1 integrin, were observed to abrogate vesicle-induction of IL-1β production and down-stream phosphorylation of Akt and c-Jun (Atay et al., 2013). This approach reveals the importance of receptor/ligand interactions in vesicle communication.

Extracellular vesicles can be internalized by recipient cells following receptor-ligand interactions and the varied assortment of bioactive molecules, derived from the cell of origin, such as proteins, bioactive lipids, and nucleic acids, can be transferred along with the proteins expressed on the vesicle surface. Extracellular vesicles may directly activate the recipient cell by acting as signaling complexes (Corrado et al., 2013; Ji et al., 2013). Extracellular vesicles derived from macrophages bind to platelets by means of the P-selectin glycoprotein ligand-1 expressed on their surface and extracellular vesicles from neutrophils expressing Mac-1 may induce platelet activation (Brouckova and Holada, 2009). Extracellular vesicles may also transfer receptors from one cell to another. Bystander B cells have been shown to rapidly acquire antigen receptors from activated B cells by membrane transfer with the resulting increase of a cell population presenting a specific antigen to CD4 T cells (Quah and O'Neill, 2005). Also, Fas ligand can be transferred from tumor cells by extracellular vesicles provoking activated T cell apoptosis (Teng et al., 2012). Moreover, extracellular vesicles may convey proteins to the cytoplasm of recipient cells, such as the cell death caspase-1 message conveyed by microvesicles derived from LPS-stimulated monocytes (Dubyak, 2012), or the tumor exosome-carried Notch ligand Delta-like 4 which inhibits Notch signaling, enhancing angiogenesis (Sheldon et al., 2010).

When released extracellular vesicles fuse with their target cells, they can transfer specific membrane components, including receptors and ligands, which can express an activated phenotype. This transfer of receptors from extracellular vesicles to target cells was demonstrated by the observation that bystander B cells acquire antigen receptors from activated B cells by membrane transfer (McLellan, 2009). This transfer allows the amplified expansion of antigen-binding B cells with the ability to present specific antigens to CD4 T cells. Extracellular vesicles can transfer the adhesion molecule CD41 from platelets to endothelial cells or to tumor cells, conferring pro-adhesive properties to the target cell (Ratajczak et al., 2006). Exosome-mediated transfer of Fas ligand from tumor cells induces apoptosis of activated T cells enabling tumor immune escape (Ichim et al., 2008). Extracellular vesicles can also be protective for tumor cells by removing molecules, such as Fas or the membrane attack complex, from their membranes.

The horizontal transfer of macromolecules and their functional consequences has been demonstrated in human gliomas (Al-Nedawi et al., 2008, 2009a). In this model, only a fraction of the cells, exhibiting a transformed phenotype, expressed the truncated epidermal growth factor receptor, EGFRvIII, associated with dysregulaed tumor growth (Al-Nedawi et al., 2009b). Al-Nedawi et al. (2008) demonstrated transfer of the oncogenic EGFRvIII from human glioma cancer cells expressing the receptor to glioma cells without the EGFRvIII via the fusion of extracellular vesicles. After transfer, the glioma cells, lacking the receptor, were transformed to express EGFRvIII-regulated genes, including VEGF, Bcl-x_L_, and p27 (Al-Nedawi et al., 2009a). Subsequent studies demonstrated that the oncogenic EGFRvIII from human squamous cell carcinoma cells was transferred via extracellular vesicles to tumor-associated endothelial cells to activate MAPK and Akt cell signaling pathways and promote endothelial VEGF expression (Al-Nedawi et al., 2009b).

Epigenetic changes have been frequently demonstrated in various tumors, resulting in regulation of gene transcription, altered proliferation, differentiation, and therapeutic resistance (Viswanathan et al., 2003; Camussi et al., 2010). Genetic information can be transferred through two proposed mechanisms: vertical gene transfer, gene exchange from parent to the next generation, and horizontal gene transfer, induced through, for example, bacteriophages or viruses. Since extracellular vesicles have been implicated as a potent source of macromolecule transfer to neighboring and distant cells, viruses and other pathogens appear to exploit this system. Extracellular vesicles are postulated to contribute to the spread of infective agents, such as human immunodeficiency virus type 1 (Lenassi et al., 2010). In macrophages receiving chemokine receptors, this can induce an increased risk of HIV infection together with resistance to apoptosis. The transfer of the chemokine (CXC motif) receptor 4 and the chemokine (CC motif) receptor 5 chemokine co-receptors for human immunodeficiency virus type I by released extracellular vesicles can enhance the entry of the virus into cell types other than the lympho-hemopoietic lineage (Izquierdo-Useros et al., 2009). In addition to transferring receptors, extracellular vesicles can transfer viruses, contained within extracellular vesicles, by the “Trojan exosome hypothesis” involving direct delivery (Izquierdo-Useros et al., 2010).

Cell-derived extracellular vesicles represent another mechanism of horizontal gene transfer. Genomic instability may be mediated by horizontal transfer of tumor-derived materials via extracellular vesicles. Horizontal transfer of macromolecules, including RNA, proteins and lipids, via extracellular vesicles has been shown in a multiple tumor systems, including: gliomas, monocytes, mast cells, and T cells (Skog et al., 2008). Tumor-derived extracellular vesicles have been shown to be capable of transferring surface components (proteins and lipids) and RNAs to monocytes. Janowska-Wieczorek et al. (2005) demonstrated that extracellular vesicles derived from murine embryonic stem cells (ESCs) could induce epigenetic reprogramming of target cells. ESC-derived extracellular vesicles were shown to improve survival of hematopoietic stem/progenitor cells, to induce up-regulation of early pluripotent and early hematopoietic markers, and to induce phosphorylation of mitogen-activated protein kinase p42/44 and Akt. ESC-derived extracellular vesicles were shown to express mRNAs for several pluripotent transcription factors that can be delivered to target cells and translated to their corresponding proteins (Koh et al., 2010). As RNase-treatment inhibited their exosome-mediated biological effect, the involvement of mRNA in the observed biological effects was suggested. Yuan et al. (2010) have shown that in addition to mRNA, extracellular vesicles can transfer microRNA to target cells. They demonstrated that extracellular vesicles derived from ESCs contain abundant microRNA and that they can transfer a subset of microRNAs to mouse embryonic fibroblasts *in vitro*. Since microRNAs are regulators of protein translation, this observation raised the possibility that stem cells can alter the expression of genes in neighboring cells by transferring exosomal microRNAs. When shed vesicles fuse with their target cells, the portion of cytosol segregated within their lumen is discharged to and integrates with the cytosol of the target cell. Because this transfer can also include transmission of specific mRNAs, it can ultimately contribute to the epigenetic and proteomic properties of target cells.

Extracellular vesicles have been proposed to re-model and educate the host environment to generate a favorable niche for tumor growth, invasion and spread of metastasis (Peinado et al., 2012). Extracellular vesicles from lung cancer cells can activate the expression of pro-angiogenic factors, including IL-8, VEGF, LIF, oncostatin M, IL-11 and MMP 9 in adjacent stromal cells, “educating” the microenvironment to support lung cancer cell metastasis (Kucharzewska et al., 2013). Extracellular vesicles released by tumors have been shown to have immunosuppressive functions, including the suppression of both T lymphocytes and natural killer cell activation (Abusamra et al., 2005; Ashiru et al., 2010). It has also been suggested that tumor cell extracellular vesicles can migrate to the lymph nodes and condition them to become more favorable environments for metastasis (Rana et al., 2013). Recent studies demonstrate that the extracellular vesicles target specific organs and their presence can initiate organ tropism of metastases. It has been postulated that microRNAs delivered by extracellular vesicles play a role in immune system regulation. An extracellular vesicle-dependent exchange of microRNAs between APC and T cells occurs at the site of immune synapses (Gutiérrez-Vázquez et al., 2013), including the exchange of genetic information between DCs and T cells through extracellular vesicles (Mittelbrunn et al., 2011).

It has been suggested that tumor cell progression could use multiple forms of extracellular vesicle-mediated communication to simultaneously affect multiple effector populations, based on release of tissue factors, immunoregulators and oncogenic molecules. Thus, the signals transferred to neighboring and distant cells via extracellular vesicles may mirror the transcriptional status of the parent cell, but due to the exosomal messenger RNA and microRNA being transferred, their consequences on the translational machinery of the target cells are extensive.

## Diagnostic application of RNA within extracellular vesicles

There are two critical roadblocks for successful long-term survival of patients with cancer. First, there are no clinically useable markers to identify early stage cancers in asymptomatic individuals or to differentiate benign from malignant disease. Second, there are no methods to evaluate the dynamic changes in tumors during therapy. Standard imaging approaches do not provide metrics of tumor-specific genetic/phenotypic changes and operative information is expensive, potentially morbid and limited by errors in topographic sampling. Lacking metrics, clinicians are left unable to target appropriate therapy to tumors in individual patients. This seriously compromises the development of novel effective therapies in addition to effective and improved use of current modalities. Circulating biomarkers have been proposed to be promising for the definitive diagnosis and monitoring treatment of various tumor types. Defining tumor-specific biomarkers has numerous advantages, such as diagnosing the disease, identifying processes that are difficult to image, predicting outcome by identifying patients at risk for therapeutic failure, defining tumor-specific molecular and pathological alterations for developing therapeutic targets and monitoring responses to acute interventions (Kim et al., 2013). Such circulating biomarkers could also serve to monitor disease progression and predicting risk of recurrence; however, circulating biomarkers are problematic and exhibit several critical issues. Free protein and nucleic acid biomarkers are extremely unstable in the circulation, thus to detect these, a high steady-state must be reached for detection, which is generally not observed except in late stage disease, minor changes over time (essential for monitoring) are difficult to quantify and these biomarkers are sensitive to sample handling. The use of exosome-associated biomarkers appears to be capable of circumventing these issues. These tumor-derived extracellular vesicles are extremely stable within the circulation, in the order of days (vs. minutes for soluble markers). In addition to serving as biomarkers of cancer, data demonstrate that these tumor-derived exosomes may mediate events associated with tumor progression and metastases.

Exosomes provide stable, disease-specific markers for detection, disease characterization, and predicting prognosis (Liang et al., 2013). Temporal changes in exosomal RNA profiles have been demonstrated to accurately predict disease recurrence and overall patient survival (Takeshita et al., 2013). The proteomic and genomic profiles of circulating exosomes provide a real-time monitor of therapeutic response, serving as a companion diagnostic. By correlating these circulating markers with the molecular characteristics and real-time clinical parameters, he has established the use of circulating exosomes as a “liquid biopsy.” In 2008, we published the initial demonstration of circulating exosomal RNA for their diagnostic use (Taylor and Gercel-Taylor, 2008). Since that time, many studies have examined the diagnostic utility of profiling total circulating microRNA in specific pathologies; however, no study, to date, has defined circulating exosome microRNA signatures derived from a single cell type. The release of exosomal RNAs exhibit features for utility as diagnostic biomarkers, as they can be detected at early stages, are present in routinely obtained biologic fluids (blood, CSF, urine, and saliva), are specifically derived from the cancer tissue, and can be easily and accurately quantified. Studies have demonstrated that extracellular vesicles are enriched in tumor-derived bioactive molecules. The level of extracellular vesicles in the peripheral blood of healthy controls has been observed to be ~10^10^/ ml of blood and may increase 3–4 fold in patients with cancer.

One issue for the use of extracellular vesicles as diagnostic markers is that they are also released by other cells associated with the peripheral blood, including lymphocytes, platelets, and endothelial cells. This has established the need for isolation of specific vesicles populations, To address this, in our initial study, extracellular vesicles of tumor origin were isolated from the blood of women with ovarian cancer using antibodies reactive with epithelial cell adhesion molecule (EpCAM). It was also shown that the level of circulating EpCAM-positive extracellular vesicles increased with disease progression (Taylor and Gercel-Taylor, 2008). Furthermore, eight microRNAs previously shown to be overexpressed in ovarian cancer were demonstrated to be present in both the tumors and the serum extracellular vesicles with a strong correlation in expression levels. This suggests that the exosomal RNA can be used to characterize the tumor without having to obtain biopsies of the tumor. A significant increase in the levels of the eight microRNAs was also seen in extracellular vesicles from patients with ovarian cancer, compared to the benign samples. This study demonstrated for the first time that the RNA in extracellular vesicles could be used as a diagnostic marker for cancer and importantly, it could be used to distinguish benign vs. malignant tumors (Taylor and Gercel-Taylor, 2008). A year later, similar results demonstrated that the microRNA in serum extracellular vesicles from lung cancer patients could be used as a diagnostic marker (Rabinowits et al., 2009). It was shown that patients with lung cancer also had more EpCAM-positive extracellular vesicles in their serum, compared to controls. The levels of 12 microRNAs previously found in lung tumor biopsies were detected in both the serum extracellular vesicles and the tumors. Again, a strong correlation in microRNA expression was found between these two sources (Rabinowits et al., 2009). These two studies showed that exosomal microRNAs, while exhibit some unique features, are generally representative of the tumor, suggesting that instead of biopsies, serum extracellular vesicles could be a relatively non-invasive route to profile a tumor.

The RNA content of serum extracellular vesicles from glioblastoma patients has also been investigated for its potential role as a biomarker. Skog et al. (2008) demonstrated mutated version of the epidermal growth factor receptor (EGFR) mRNA, *EGFRvIII*, detected in 47% of the tumors in glioblastoma patients, could also be detected in serum-derived extracellular vesicles in 28% of the patients. This mRNA was not detected in the serum extracellular vesicles from healthy controls. After surgical removal of the tumor, the mRNA could no longer be detected in the patients' peripheral circulation, demonstrating the tumor as the source of the extracellular vesicles (Skog et al., 2008). This study showed that the vesicle-associated mRNA could serve as biomarkers, however, the correlation between mRNA expression in the extracellular vesicles and tumor was weaker than that observed in microRNA studies. Most critically, this study demonstrated that mutations in the tumor could be identified within RNA isolated from extracellular vesicles.

Extracellular vesicles are released by many cells, including as reticulocytes, dendritic cells, B cells, T cells, mast cells, macrophages, epithelial cells, and tumor cells (Gallo et al., 2012). Within mast cell lines, 121 microRNAs, including miR-1, miR-15, miR-16, miR-17, miR-18, miR-181, miR-375, lin-4 and let7, were demonstrated within their released extracellular vesicles (Valadi et al., 2007). Other microRNAs, such as miR223 expressed in IL-4-activated macrophages, miR451 in dendritic cells and miR335 in T cells, have been demonstrated in extracellular vesicles from normal cell types (Mittelbrunn et al., 2011; Yang et al., 2011). Based on these observations, most extracellular microRNAs within the peripheral circulation are primarily in extracellular vesicles (Gallo et al., 2012).

## Conclusions

While the use of extracellular vesicles as biomarkers in the clinical setting remains years away, the findings presented within this review demonstrate their significant diagnostic potential. This overview of the RNA cargoes of extracellular vesicles reveals their ability to distinguish benign ovarian tumors from malignant disease. Further, we can obtain and analyze the microRNA profiles of lung and ovarian tumors prior to surgery, we can identify the mutation status of tumors based on a blood specimen, and we can predict patient survival and risk of recurrence.

In the 5 years since our initial demonstration of the diagnostic utility of RNA profiles from extracellular vesicles, new and sensitive techniques have been developed, including deep sequencing and focused microarrays and real time PCR, which can expand the information from transcriptome from the tumor-derived extracellular vesicles. The vesicle platform for diagnostics provides a multiplex approach to studying biomarkers relying on a single marker. This additional genetic information will extend the potential of using circulating vesicular RNAs as biomarkers in diagnostics, but provides biomarkers for with patient stratification, selection of personalized therapies, companion diagnostics and for monitoring therapeutic responses. As early detection is essential for improved patient survival, the utility of cargo analyses of extracellular vesicles will be particularly valuable in identifying asymptomatic patients and stratify patient populations. While microRNA expression profiles have been the primary RNA population investigated, current findings demonstrate the presence of other RNAs that may be important in malignant properties linked with progressive cancer. Current and future studies are addressing the ability of vesicle-associated RNAs to identify the risk for developing cancer, monitoring its progression, and predict its prognosis. The development of their vesicular RNA markers may also serve as valuable tools in companion diagnostics, for assessing clinical trials and defining therapeutic options.

### Conflict of interest statement

The authors declare that the research was conducted in the absence of any commercial or financial relationships that could be construed as a potential conflict of interest.
